# Functional Locomotor Consequences of Uneven Forefeet for Trot Symmetry in Individual Riding Horses

**DOI:** 10.1371/journal.pone.0114836

**Published:** 2015-02-03

**Authors:** Nathan Wiggers, Sandra L. P. Nauwelaerts, Sarah Jane Hobbs, Sophie Bool, Claudia F. Wolschrijn, Willem Back

**Affiliations:** 1 Department of Equine Sciences, Faculty of Veterinary Medicine, Utrecht University, Utrecht, The Netherlands; 2 Department of Biology, University of Antwerp, Antwerp, Belgium; 3 Centre for Applied Sport and Exercise Sciences, University of Central Lancashire, Preston, United Kingdom; 4 Department of Pathobiology, Faculty of Veterinary Medicine, Utrecht University, Utrecht, The Netherlands; 5 Department of Surgery and Anaesthesiology, Faculty of Veterinary Medicine, Ghent University, Merelbeke, Belgium; Faculty of Animal Sciences and Food Engineering, University of São Paulo, Pirassununga, SP, BRAZIL

## Abstract

Left-right symmetrical distal limb conformation can be an important prerequisite for a successful performance, and it is often hypothesized that asymmetric or uneven feet are important enhancing factors for the development of lameness. On a population level, it has been demonstrated that uneven footed horses are retiring earlier from elite level competition, but the biomechanical consequences are not yet known. The objectives of this study were to compare the functional locomotor asymmetries of horses with uneven to those with even feet.

Hoof kinetics and distal limb kinematics were collected from horses (n = 34) at trot. Dorsal hoof wall angle was used to classify horses as even or uneven (<1.5 and >1.5° difference between forefeet respectively) and individual feet as flat (<50°), medium (between 50° and 55°) or upright (>55°). Functional kinetic parameters were compared between even and uneven forefeet using MANOVA followed by ANOVA. The relative influences of differences in hoof angle between the forefeet and of absolute hoof angle on functional parameters were analysed using multiple regression analysis (P<0.05).

In horses with uneven feet, the side with the flatter foot showed a significantly larger maximal horizontal braking and vertical ground reaction force, a larger vertical fetlock displacement and a suppler fetlock spring. The foot with a steeper hoof angle was linearly correlated with an earlier braking-propulsion transition.

The conformational differences between both forefeet were more important for loading characteristics than the individual foot conformation of each individual horse. The differences in vertical force and braking force between uneven forefeet could imply either an asymmetrical loading pattern without a pathological component or a subclinical lameness as a result of a pathological development in the steeper foot.

## Introduction

A poor distal limb conformation in horses is usually related to a predisposition to lameness [1–5] and early retirement from competition [6–8]. Poor distal limb conformation would also include unevenness, where the two forefeet differ in shape, size and hoof angle [9] commonly referred to as uneven feet. Uneven feet indeed are considered to be an important factor in the development of lameness [4,5], are associated with side preferences during locomotion [10] and are also reported to lead to early retirement in elite level sports horses, show jumpers more likely than dressage horses [11].

To date, the development of uneven feet has been linked to postural and loading preferences during standing [9, 12, 13] and pain avoidance [4,5]. Uneven feet were found in horses with a lateral preference during asymmetrical standing posture, such as grazing [9] and the condition was particularly evident in foals and mature long-legged horses with short necks, as they have to spread their limbs further apart [14]; this phenomenon has even been investigated at a Warmblood population level [15]. Along with posture, geometry of the foot was found to influence loading patterns. Flatter feet were reported to have larger moment arms from the centre of pressure at the ground to the proximal and distal interphalangeal joints compared to upright feet [9, 12,13]. Hoof growth between shoeing intervals may exacerbate the condition further, as the distal interphalangeal joint moment arm in flat feet was found to increase disproportionately compared to in upright feet [13]. During an 8-week shoeing interval, the toe was found to grow further, while the heels were subjected to wear. Heel wear was attributed to the interaction of the heels with the shoe due to the hoof mechanism, in combination with increased heel pressure from a low-heeled foot conformation. Pain has also been attributed to the development of uneven feet, as it was surmised that pain in the distal limb was expected to lead to heel unloading and thus the development of a more upright hoof [4,5]. However, horses showing signs of unilateral palmar foot pain are often more lame at the trot, whereas when a developmental mechanical asymmetry is involved, lameness merely becomes more evident at the walk [9, 12, 13].

Although these studies provide important information about asymmetric posture and loading patterns that may develop unevenness, they principally address differences during standing. Little is known, however, about any functional asymmetries that may be present in horses with uneven feet during locomotion. Earlier studies that considered hoof angle variation effects on locomotion principally investigated bilateral and only artificially induced changes during trotting, as trot is a symmetrical gait used for lameness assessment. In relation to timing, a later onset of breakover and a later transition from longitudinal braking to propulsion was found in flatter footed limbs [16], although stance duration was reported to be relatively independent of induced changes in foot conformation [16–19]. Similarly, changes in timing variables during locomotion were not evident between shoeing intervals, but instead were reflected in distal limb joint angle differences [9, 13]. Metacarpophalangeal joint (MCPJ) extension was reported to have a strong relationship with peak vertical force production at trot [20], so maximal extension may provide clues in relation to changes in loading pattern and limb stiffness with differences in hoof angle. To date, differences in maximal MCPJ extension with the application of a heel wedge were either not found to be significant [18,21], or were found to reduce maximal extension [22] compared to no heel wedge. From these studies it is unclear whether naturally developed unevenness will influence loading patterns in relation to peak force production, timing or impulse variables during trotting.

**Figure 1:**
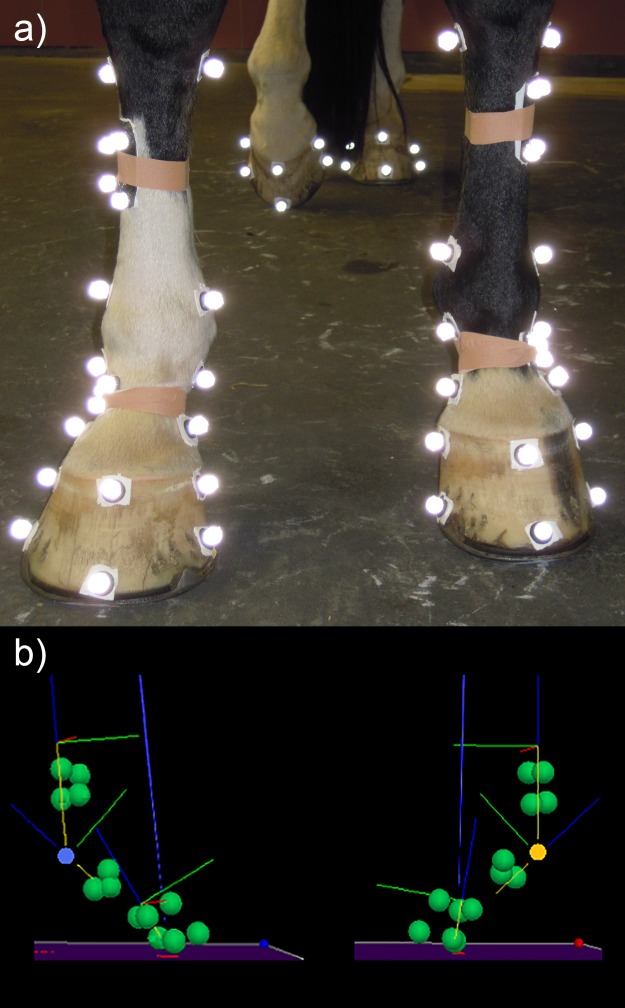
Photograph and three-dimensional reconstruction of one uneven footed horse showing the marker set used in the study. a) A photograph showing the anatomical and tracking markers used for the study. The photograph illustrates the definition of a horse with uneven feet where the right forelimb has a lower hoof angle (LHA) and the left forelimb has a higher hoof angle (HHA). b) An example of the functional consequences of unevenness in one horse with a difference in dorsal hoof wall angle of 8 degrees. Three-dimensional reconstruction of the left and right forelimbs in the position of maximum vertical MCPJ displacement. The blue and yellow circles show the position of the MCPJ and the blue line originating from the centre of the foot is the resultant force vector. The hoof on the left is the lower hoof angle (LHA; blue: RF) and the hoof on the right is the higher hoof angle (HHA; yellow: LF).

The aim of this study was to compare the functional kinematic and kinetic locomotor asymmetries of horses with uneven (as shown in Fig. 1) to those with even feet. Functional differences between feet with differing dorsal hoof wall angles rather than differences associated with individual feet in specific angle ranges were found.

## Materials and Methods

### Horses

Thirty-four riding horses of different breeds, mean mass 557 ± 77 kg (mean ± s.d.) and mean age 12 ± 5 years were included in the study. Horses were graded at walk and trot independently on a hard surface and in a straight line by an experienced clinician using a modified American Association of Equine Practitioners (AAEP) lameness scale, in which separate scores from 0–5 were recorded for walk and for trot (Table 1). Horses were not scored on circles, as asymmetry in loading and movement patterns during locomotion on a circle has been reported previously [23,24]. The forefeet were classified based on the difference in measured dorsal hoof wall angle (Fig. 1). Differences larger than 1.5 degrees between left and right were considered uneven based on discriminant analysis. Prior to data collection, all horses were habituated to the data collection area. Horses were all routine patients of the Equine Clinic of Utrecht University with an informed consent of their owners. Thus, it was considered that there was no additional need for Animal Care and Ethics Committee approval according to Dutch law.

**Table 1:** Number of even and uneven footed horses and modified AAEP lameness scores 0/5 trot for walk and for trot separately.

Lameness score	Even (N)	Uneven (N)
0/5 walk, 0/5 trot	7	19
1/5 walk, 0/5 trot	0	8
	**7**	**27**

Grade 0 = sound, Grade 1 = lameness is difficult to observe and not consistently apparent, Grade 2 = lameness is shown with a consistently apparent minimal head nodding, Grade 3 = lameness is clearly recognized from apparent head nodding, Grade 4 = lameness is obviously recognized from a marked head nodding, Grade 5 = lameness is characterized by non-weight bearing in motion.

### Data collection

The feet were cleaned with isopropyl alcohol. Retro-reflective markers were attached to the skin at the dorsal edge of the head of the medial and lateral second and fourth metacarpal bones, the proximal attachment site of the medial and lateral collateral ligaments of the MCPJ, the medial and lateral tuberosities of the proximal aspect of the proximal or first phalanx (P1), the medial and lateral tubercle at the attachment site of the medial and lateral collateral ligament of the proximal interphalangeal joint [25,26], and the dorsal hoof wall, one at the coronary band and the other at the distal border of the hoof in line with the so called foot axis. These markers were used to define the third metacarpal bone (MC3) segment, P1 segment and hoof angle. A cluster of four markers mounted on a rigid carbon fibre shell was attached to the skin overlying the dorsolateral mid-shaft of the MC3. A cluster comprising three tracking markers was placed over the dorsolateral aspect of the P1. The markers on each cluster were referenced to the anatomical markers in the standing trial and then used to track the movement of the segments during locomotion based on the Calibrated Anatomical Systems Technique [27,28] (Fig. 1).

Kinematic data were captured with an eight-camera infrared motion capture system (Qualisys Oqus 3+) at 250 Hz. A standing trial with the horse standing square was captured initially with both anatomical and cluster markers in place. The anatomical markers were then removed. A handler led each horse at trot in a straight line at a consistent velocity over a force platform (Kistler Z4852C, 60 × 90 cm), which captured data at 1000 Hz. Summed fore and hindlimb impulses for each diagonal were compared post testing to ensure velocity was consistent between trials. The force plate and the surrounding running track were covered with a rubber mat. A minimum of 3 sufficient measurements were recorded and analyzed for each forelimb.

### Data Processing

Kinematic data from the standing and motion trials were identified (Qualisys Track Manager 2.5) and exported to motion analysis software (Visual 3D 4.96). A model was developed from the standing trial, which identified the functional joint centre for the MCPJ. A longitudinal axis for the MC3 was defined by the medial and lateral anatomical markers positioned proximally and distally. The flexion-extension axis of the MCPJ was then computed from the motion trials using a custom method [29]. The functional joint center of the MCPJ was then located at the intersection of the flexion-extension axis and the longitudinal axis of the MC3. Hoof angle was defined as the angle of the line joining the proximal and distal marker at the dorsal hoof wall. Every individual foot was categorized as upright (hoof angle > 55°), medium (hoof angle between 50° and 55°) or flat (hoof angle < 50°). This categorization was based upon the normal hoof angle range in Warmblood sports horses of 50° to 55° as reported earlier [5, 13, 30]. The absolute difference in hoof angle between forefeet was calculated for each horse and this was used to categorize each horse as even or uneven (1.5 degrees).

**Figure 2:**
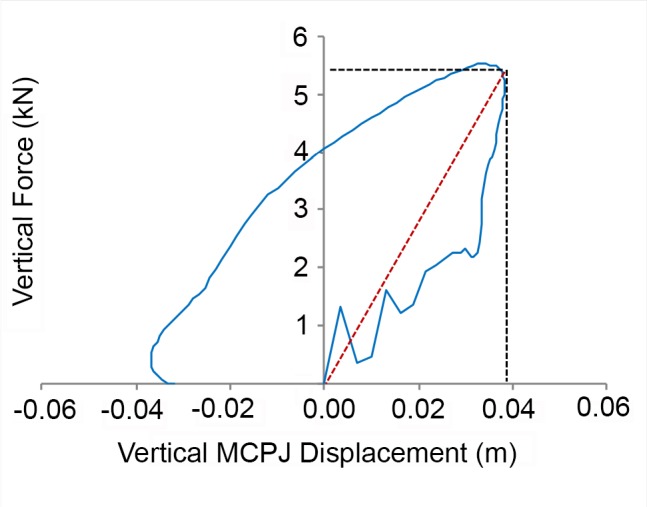
Calculation of effective vertical stiffness. Vertical ground reaction force plotted against vertical displacement of the MCPJ during the stance phase (blue line) to illustrate how effective vertical stiffness was calculated. Stiffness was determined from the magnitude of the vertical force (black dotted horizontal line) at maximum vertical MCPJ displacement (black dotted vertical line), so the slope of the red dotted line represents the effective vertical stiffness.

Cluster markers in the motion trials were filtered using a critically damped 4^th^ order Butterworth filter [31] with a cut-off frequency of 12 Hz from which MCPJ displacement was determined. A 12 Hz cut-off frequency was used as >90% of the signal power was below this frequency. Ground reaction forces were down-sampled to 250 Hz and extracted together with MCPJ displacement. These data were then imported into custom-made calculation software (Matlab 8.0), which was used to calculate functional parameters. Functional force parameters were vertical, braking and propulsive impulse, peak and the relative time to peak vertical, braking and propulsive force, stance duration and the relative time to the transition from braking to propulsion. Force variables were normalized to body mass and time variables as a percentage of the stance phase (100%). Vertical force was plotted against vertical MCPJ displacement and the following additional functional parameters were extracted, being maximal vertical displacement of the MCPJ and stiffness (slope of the force-displacement curve from the start of the stance phase to the point of maximal vertical displacement of the fetlock joint; Fig. 2). Stiffness was calculated based on the previously described ‘effective vertical stiffness’ [32,33].

### Data Analysis

A number of 6 out of 19 functional parameters were transformed to meet the assumption of normality, using the ladder of powers transformation [34]. All analyses were performed in commercially available statistical software (SPSS 21.0) software and results were considered significant if P<0.05.

### Relationship between unevenness and functional parameters

To test if uneven footed horses showed more functional asymmetries between the forefeet than even footed horses, the following procedure was performed. For each horse, the foot with the highest hoof angle was classified as highest hoof angle (HHA) foot, and the foot with the lowest hoof angle was classified as lowest hoof angle (LHA) foot. Full factorial MANOVA followed by ANOVA tests were conducted on functional parameters, separately for horses with even and for those with uneven feet. Foot category (LHA/HHA) was used as fixed factor and horse was used as random factor.

### Relationship between individual foot conformation and functional parameters

In order to test whether the functional parameters were different between foot categories (upright, medium, flat), full factorial MANOVA, followed by ANOVA were used. Foot category (upright/medium/flat) was used as fixed factor and horse was used as random factor. Scheffé’s post hoc test was used to compare foot categories for any variables that were found significant for the main effect foot category.

### Relative weight of conformational differences between the forefeet and of individual foot conformation

To evaluate the relative weight of the conformational differences between the feet and of individual foot conformation on the significant results of the analyses multiple linear regression analyses were performed. For the functional parameters of each horse, mean values were calculated per foot. The functional parameters were tested individually by multiple regression analysis. Difference in hoof angle and absolute hoof angle were used as independent variables to test their relative influence on the functional parameter.

## Results

No significant difference was found between diagonals for summed fore and hindlimb impulses (P = 0.923).

### Relationship between unevenness and functional parameters

**Figure 3:**
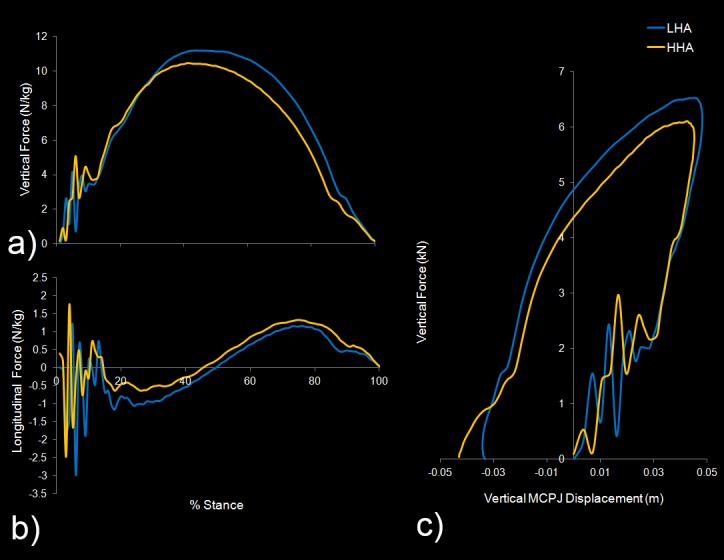
Graphical data of the functional consequences of unevenness in one horse with a difference in dorsal hoof wall angle of 8 degrees. a) Vertical force (N/kg) during the stance phase showing the difference in peak force and time to peak force between limbs. b) Longitudinal force (N/kg) during the stance phase showing the difference in braking and propulsive force and the time of the transition from braking to propulsion. c) Stiffness curve for each limb highlighting how maximum MCPJ displacement occurs prior to peak vertical force in the LHA limb.

Relationships between functional parameters and unevenness are shown in Table 2. Functional parameters that were found to be significantly different (P<0.05) for horses with uneven feet were peak vertical and peak braking force, braking impulse, time to the transition from braking to propulsion, stiffness and vertical MCPJ displacement (see Fig. 3). None of these functional parameters were found to be significantly different (P<0.05) for horses with even feet. No other functional parameters were found to be significantly different (P<0.05) for horses with uneven feet.

**Table 2:** Comparison of functional parameters of the lowest hoof angle (LHA) foot with those of the highest hoof angle (HHA) foot in horses with uneven feet as well as in horses with even feet.

		**Uneven**	**Even**
**Peak Vertical Force (N/kg)**	***P*-value**	**0.026**	**0.499**
	Mean LHA [95% CI]	**10.874** [10.791–10.958]	**10.838** [10.700–10.975]
	Mean HHA [95% CI]	**10.624** [10.543–10.705]	**10.748** [10.611–10.886]
**Peak Braking Force (N/kg)**	***P*-value**	**0.017**	**0.551**
	Mean LHA [95% CI]	**0.546** [0.521–0.571]	**0.552** [0.487–0.620]
	Mean HHA [95% CI]	**0.444** [0.423 0.466]	**0.600** [0.533–0.672]
**Peak Propulsive Force (N/kg)**	***P*-value**	**0.197**	**0.344**
	Mean LHA [95% CI]	**0.789** [0.772–0.807]	**0.914** [0.871–0.959]
	Mean HHA [95% CI]	**0.817** [0.800–0.835]	**0.856** [0.816–0.899]
**Vertical Impulse (Ns/kg)**	***P*-value**	**0.247**	**0.071**
	Mean LHA [95% CI]	**2.076** [2.061–2.091]	**2.087** [2.063–2.111]
	Mean HHA [95% CI]	**2.036** [2.022–2.050]	**2.053** [2.029–2.077]
**Braking Impulse (Ns/kg)**	***P*-value**	**0.025**	**0.477**
	Mean LHA [95% CI]	**0.051** [0.048–0.053]	**0.049** [0.044–0.055]
	Mean HHA [95% CI]	**0.041** [0.039–0.043]	**0.057** [0.051–0.064]
**Propulsive Impulse (Ns/kg)**	***P*-value**	**0.068**	**0.251**
	Mean LHA [95% CI]	**0.085** [0.082–0.087]	**0.103** [0.097–0.110]
	Mean HHA [95% CI]	**0.091** [0.088–0.093]	**0.094** [0.088–0.100]
**Relative Time to Peak Vertical Force (% stance)**	***P*-value**	**0.865**	**0.308**
	Mean LHA [95% CI]	**45.4** [45.0–45.9]	**44.8** [44.0–45.6]
	Mean HHA [95% CI]	**45.4** [45.0 45.8]	**45.3** [44.5–46.2]
**Relative Time to Peak Braking Force (% stance)**	***P*-value**	**0.755**	**0.768**
	Mean LHA [95% CI]	**27.2** [26.7–27.7]	**28.0** [27.0–29.0]
	Mean HHA [95% CI]	**27.1** [26.6–27.5]	**27.8** [26.7–28.8]
**Relative Time to Peak Propulsive Force (% stance)**	***P*-value**	**0.382**	**0.238**
	Mean LHA [95% CI]	**72.7** [72.4–73.0]	**72.1** [71.5–72.8]
	Mean HHA [95% CI]	**72.3** [72.0 72.6]	**73.1** [72.4–73.8]
**Relative Time from Braking to Propulsion (% stance)**	***P*-value**	**0.034**	**0.178**
	Mean LHA [95% CI]	**47.3** [46.7–47.9]	**45.5** [44.2–46.8]
	Mean HHA [95% CI]	**45.2** [44.6–45.8]	**48.2** [46.9–49.5]
**Stance Duration (s)**	***P*-value**	**0.905**	**0.584**
	Mean LHA [95% CI]	**0.319** [0.316–0.323]	**0.324** [0.317–0.330]
	Mean HHA [95% CI]	**0.320** [0.316–0.323]	**0.320** [0.313–0.327]
**Maximum MCPJ Displacement (m)**	***P*-value**	**0.006**	**0.325**
	Mean LHA [95% CI]	**0.049** [0.048–0.050]	**0.046** [0.044–0.048]
	Mean HHA [95% CI]	**0.045** [0.044–0.046]	**0.044** [0.042 0.047]
**Stiffness (kN/m)**	***P*-value**	**0.008**	**0.531**
	Mean LHA [95% CI]	**120.1** [117.1–123.0]	**123.3** [116.6–130.0]
	Mean HHA [95% CI]	**127.8** [124.9–130.6]	**126.8** [120.0–133.5]
**Vertical force at Max MCPJ Displacement (N/kg)**	***P*-value**	**0.161**	**0.493**
	Mean LHA [95% CI]	**10.32** [10.22–10.43]	**10.21** [10.05–10.38]
	Mean HHA [95% CI]	**10.12** [10.02–10.22]	**10.12** [9.96–10.29]

The table shows ANOVA results for the discriminant function classification. For each analysis, P-values, means and 95% confidence interval [CI] are presented.

### Relationship between individual foot conformation and functional parameters

None of the functional parameters were significantly different (P<0.05) between feet categorized as flat, medium or upright, see Table 3.

**Table 3:** Functional differences between feet categorized as flat, medium or upright.

		***P*-value**	**Flat Mean [95% CI]**	**Medium Mean [95% CI]**	**Upright Mean [95% CI]**
**Peak Force (N/kg)**	**Vertical**	0.256	**10.55** [10.465–10.644]	**10.835** [10.748–10.922]	**10.804** [10.690–10.918]
	**Braking**	0.057	**0.521** [0.491–0.553]	**0.531** [0.502–0.562]	**0.430** [0.395–0.466]
	**Propulsive**	0.516	**0.751** [0.732–0.770]	**0.874** [0.852–0.896]	**0.862** [0.835–0.891]
**Impulse (Ns/kg)**	**Vertical**	0.316	**2.069** [2.053–2.086]	**2.030** [2.014–2.046]	**2.039** [2.018–2.059]
	**Braking**	0.057	**0.049** [0.046–0.053]	**0.048** [0.045–0.051]	**0.039** [0.036–0.042]
	**Propulsive**	0.249	**0.083** [0.080–0.086]	**0.092** [0.089–0.095]	**0.096** [0.092–0.100]
**Relative time to (% Stance)**	**Peak Vertical Force**	0.546	**45.7** [45.2–46.1]	**45.4** [45.0–45.9]	**45.7** [45.1–46.3]
	**Peak Braking Force**	0.504	**27.4** [26.9–27.9]	**27.0** [26.5–27.5]	**27.4** [26.8–28.1]
	**Peak Propulsive Force**	0.686	**73.1** [72.7–73.4]	**72.4** [72.0–72.7]	**72.9** [72.4–73.3]
	**Braking to Propulsion**	0.182	**47.4** [46.7–48.1]	**46.0** [45.3–46.7]	**45.0** [44.1–45.9]
**Stance Duration (s)**	**Stance duration**	0.586	**0.326** [0.322–0.330]	**0.312** [0.309–0.316]	**0.315** [0.310–0.319]
**Stiffness Functional Parameters**	**Max MCPJ Displacement (m)**	0.233	**0.049** [0.047–0.050]	**0.045** [0.043–0.046]	**0.048** [0.046–0.049]
	**Stiffness (kN/m)**	0.383	**119.6** [116.2–123.1]	**125.9** [122.7–129.1]	**127.8** [123.4–132.2]
	**Vertical force at Max MCPJ Displacement (N/kg)**	0.346	**9.989** [9.873–10.106]	**10.223** [10.114–10.331]	**10.530** [10.382–10.679]

The table shows ANOVA results. For each analysis, P-values, mean (shown in bold) and 95% confidence intervals [CI] (shown in square brackets) are presented for flat, medium and upright feet.

### Relative weight of conformational differences between the forefeet and of individual foot conformation

From multiple regression analysis only the time to the transition from braking to propulsion showed a significant moderate association with absolute hoof angle and difference in hoof angle (multiple R = 0.411, P = 0.002), see Table 4. For this parameter, the standardized regression coefficient (Beta) of the difference in hoof angle was 1.6 times larger in magnitude (−0.289) than that of the absolute hoof angle (−0.180). For the peak vertical force, the multiple regression models was close to significance (P = 0.051). For vertical MCPJ displacement, the standardized regression coefficient (Beta) of the difference in hoof angle was significant (Beta = −0.284, P = 0.044). However, the entire model was not significant. There were no significant (P<0.05) linear relationships for the other functional parameters.

**Table 4:** Linear relationship of hoof angle and difference in hoof angle with functional parameters.

	**Entire model**			**Independents**		
	**Multiple *R***	***R*^2^**	***P*-value**		**Beta**	***P*-value**
**Braking to Propulsion (% Stance)**	0.411	0.169	0.002	Absolute hoof angle	−0.180	0.177
				Difference hoof angle	−0.289	0.032
**Peak Vertical Force (N/kg)**	0.296	0.087	0.051	Absolute hoof angle	0.292	0.038
				Difference hoof angle	−0.306	0.030
**Maximum MCPJ Displacement (m)**	0.272	0.074	0.088	Absolute hoof angle	0.027	0.848
				Difference hoof angle	−0.284	0.044

The table shows the significant (P>0.05) results of the multiple linear regression analysis.

## Discussion

This study investigated the functional consequences of uneven feet during trotting in sound horses with even and uneven feet. The relative timing of the transition from braking to propulsion was found to be later in the LHA foot in uneven footed horses, which in part supports our hypotheses and is accordance with the effect of an acute hoof wall angulation kinematically reported earlier [16]. Greater limb stiffness and reduced vertical MCPJ displacement in the HHA foot were also in support of kinematic adaptations as reported earlier in the lame limb [20]. Nevertheless, the recorded differences in peak vertical force in uneven footed horses were not expected, as the horses were graded as sound under the same conditions by a clinician [9, 12, 13]. In addition, individual foot confirmation was not found to be as important as the difference between feet [9,16–19].

### Relationship between unevenness and functional parameters

Results of this study indicate that there was a difference in function between limbs of uneven footed horses. Since our data were limited to visually non-lame horses at trot, we expected that the vertical forces would not differ between the forefeet. It has been reported that the human ability to detect asymmetrical movement is limited, as movement asymmetries below 25% in the hind limbs remain undetectable to the observer [35]. Although agreement in forelimb lameness between clinicians is reported to be higher [36], even a highly trained clinician would not be expected to detect such a subtle alteration in loading. In contrast, subtle lameness can be detected by left-right asymmetries in peak vertical forces using a force platform, with the lame limb showing the lower peak force [37–40].

The reduction in the peak vertical force of the HHA foot in our study was lower than the reduction of 4% reported for a subtle visually detectable lameness [40]. This could imply an early, subclinical sign of lameness developing in the HHA foot. This was supported by the fact that of the 27 uneven footed horses that were analyzed at trot, 8 were slightly lame in the HHA foot at walk, although this was considered to be a mechanical lameness [9, 12, 13]. In addition, it is conceivable that had the horses been observed moving in circles, they may actually have exhibited lameness. The question remains, however, whether the vertical force distribution between the uneven feet in the current study is related to an asymmetrical loading pattern without a pathological component or to a subclinical lameness as a result of a pathological development. In depth clinical, biomechanical and radiological monitoring over time is needed for a better understanding of the existence and direction of the link between pathological changes and asymmetrical loading due to uneven feet.

To remain at steady state trot, the braking and propulsive impulses over a stride must balance otherwise acceleration or deceleration would occur. As such the decreased braking force and braking impulse in the HHA foot may either be compensated for by increased braking forces in the contralateral forefoot, or by decreased propulsive forces in the contralateral hind foot. These effects have already been demonstrated in lame horses [38–,39]. Therefore, in horses with uneven feet, the smaller peak braking force and braking impulse in the HHA foot compared to the LHA foot could imply a subtle, visually undetectable lameness at trot, which supports the lower results for peak vertical force. Alternatively, the larger braking impulse in the LHA foot could indicate that the LHA foot was sliding more during ground contact compared to the HHA foot. This could be tested in the future by comparing the slip distance between the LHA and the HHA foot.

The hypothesis that the transition from braking to propulsion occurs later in the LHA foot compared to the HHA foot of uneven footed horses was supported by the linear positive correlation between difference in hoof angle and the timing of the transition. These findings can be associated with two mechanisms. Firstly, the later transition from braking to propulsion in the LHA feet could be related to the prolonged breakover time of hooves with a relatively long toe and a low hoof angle [16]. It takes longer for the center of mass to rotate over the flat-footed limb, leading to a later onset of breakover and a later transition from braking to propulsion. Secondly, horses with a low hoof angle show a more pronounced toe-first landing [16], which could lead to a later onset of complete hoof stabilization and breakover. Indeed, a flatter hoof landing results in a shorter duration of events after first ground contact, with a higher vertical and horizontal loading rate and a shorter braking phase [41].

In this study, horses with uneven feet showed a less stiff limb spring in the LHA foot than in the HHA foot from foot strike to maximum vertical MCPJ displacement. This is most likely caused by differences in the quality of the spring-like distal limb tissues, in particular the suspensory ligament, and the deep and superficial digital flexor muscles and tendons [42,43]. Differences in heel expansion could also play a role. The differences in stiffness between the uneven forefeet are less likely the result of possible differences in the moment arms around the distal limb joints due to the asymmetric foot conformation, since stiffness was not significantly different between flat, medium and upright feet. Although it is still unknown which of the distal limb structures could cause the asymmetry in stiffness, we defined an objectively measurable parameter to quantify the differences in MCPJ movement that are clinically observed at walk. Radiological, ultrasonographic or MR scan, and biochemical evaluation of the distal limb tissues of uneven footed horses, with special attention to the suspensory apparatus and the superficial and deep digital flexor tendons will add to the understanding of the etiology.

The timing of maximum MCPJ extension in relation to force development may also influence limb stiffness, due to the visco-elastic nature of the tissues under load. Vertical forces at the moment of maximum vertical fetlock displacement were not significantly different between the forefeet of horses with uneven feet, in contrast to peak vertical forces, which may be why head nodding is not observed. As peak vertical force was larger in the LHA foot, maximum vertical MCPJ displacement and the force at that time must have occurred earlier in the stance phase. This, in combination with the larger vertical MCPJ displacement in the LHA foot would suggest a higher vertical MCPJ velocity was reached in the LHA foot. This, in fact, could be the clinically observed asymmetry in fetlock movement in uneven footed horses.

### Relationship between individual foot conformation and functional parameters

Individual foot conformation was less important for biomechanical characteristics than the conformational differences between the forefeet, since none of the functional parameters were associated with foot category or linearly correlated with absolute hoof angle.

Stance duration and the timing of the force peaks were not different between flat, medium or upright feet, which supported the findings of previous studies [9,16–19]. This implies that stance duration and other temporal characteristics are independent of the individual foot conformation and are quite strictly controlled by the neuromuscular system. On the other hand, the fact that these temporal variables did not differ between the foot categories could be caused by between-horse variability in preferred speed.

As expected, the conformational categories showed no differences in vertical ground reaction force and this was in line with the previously found unaltered peak vertical forces after application of a 6° heel wedge [21].

The transition from braking to propulsion occurs earlier in upright feet, while flat feet show a later transition considering individual foot conformation. Based upon the reported toe-first landing in flat feet [16], one might expect a prolonged braking phase in flatter feet [41]. The fact that this idea was not supported by our findings could indicate that the different foot categories in the current study showed no differences in hoof landing pattern. Moreover, the longer breakover duration in flat feet found in previous studies [2,16,44], did not lead to an altered shape of the fore-aft force profile in the current study. Unlike our study, most previous studies are based on artificially induced changes in hoof angle. Changes in hoof angle within the animal rather than conformational differences in hoof angle between horses may therefore be more influential in producing altered longitudinal force patterns. These interpretations indeed have to be taken into account at clinical health and studbook breeding soundness examinations [45,46].

The conformation of the individual foot was not associated with the stiffness of the limb and the vertical displacement of the fetlock. Results from previous studies on the extension of the fetlock joint after the application of a heel wedge were conflicting. No significant effect of a 6° heel wedge on the maximal MCPJ extension was found at trot [18, 21], which is in line with our findings. In contrast, a significant reduction in maximal MCPJ extension after heel elevation with a 5° wedge at trot has also been demonstrated, although this was thought to be associated with a weight shift to the hind limbs [22]. Since the current study investigated vertical MCPJ displacement instead of MCPJ extension, a possible compensatory effect of the interphalangeal joints on a reduced MCPJ extension cannot be ruled out. This seems unlikely, however, since Chateau *et al.* (2006) [18] showed that a 6° heel wedge caused an increase in maximal flexion and a decrease in maximal extension of the proximal and distal interphalangeal joints at trot. A more detailed analysis of discrete stiffness during loading together with joint kinematics may be necessary to fully explain these findings.

Finally, in this study unevenness was defined as a difference of 1.5 degrees using a discriminant function analysis on several anatomical measurements, which provided a statistical definition of the two groups. Hoof angle was measured using a three-dimensional motion capture system, which was capable of producing repeated measurement to within 0.15 degrees. As hoof angles may be defined in a number of ways in a clinical setting that may not produce the same accuracy [47], our classification should be used with some caution when using other methods. Further work is needed to define unevenness using a larger population and clinical tools.

## Conclusions

This study showed that the conformational differences between the forefeet seem to be more important for loading characteristics than the individual foot conformation. The recorded differences in vertical and braking force between uneven forefeet could imply either an asymmetrical loading pattern without a pathological component or a subclinical lameness as a result of a pathological development in the steeper foot, even though these kinetic differences were smaller than those reported for a subtle lameness.
